# Poor Prognostic Factors in Surgically Treated Habitual Patellar Dislocation in Children and Adolescents

**DOI:** 10.3390/children13010068

**Published:** 2025-12-31

**Authors:** Alexandru Ulici, Mihai-Codrut Dragomirescu, Sorina-Mariana Mocanu, Alexandru Herdea

**Affiliations:** 1 11th Department of Pediatric Orthopedics, Carol Davila University of Medicine and Pharmacy, Bd. Eroii Sanitari Nr. 8, 050474 Bucharest, Romania; alexandru.ulici@umfcd.ro (A.U.); alexandru.herdea@umfcd.ro (A.H.); 2 Pediatric Orthopedics Department, Grigore Alexandrescu Children’s Emergency Hospital, 011743 Bucharest, Romania; sorina.mocanu@stud.umfcd.ro

**Keywords:** habitual patellar dislocation, pediatric patellofemoral instability, trochlear dysplasia, patella alta, ligamentous hyperlaxity

## Abstract

**Background/Objectives:** Habitual patellar dislocation is a rare but debilitating form of patellofemoral instability in children and adolescents, frequently associated with underlying anatomical abnormalities and ligamentous laxity. Despite multiple surgical techniques, recurrence and suboptimal functional recovery remain concerns. This study aimed to identify the demographic, clinical, and imaging factors associated with postoperative recurrence and poorer functional outcomes in pediatric patients surgically treated for habitual patellar dislocation. **Methods:** A retrospective cohort study was conducted on pediatric patients treated between 2016 and 2024 for habitual patellar dislocation. Inclusion criteria required age ≤ 18 years, a minimum 12-month follow-up, and complete imaging documentation. Clinical evaluation included the Beighton hyperlaxity score, lower-limb alignment, and Lysholm Knee Score. Imaging parameters assessed patellar height (Caton–Deschamps Index), trochlear dysplasia, patellar tilt, patellar subluxation, genu valgum, and tibial tubercle–trochlear groove (TT–TG) distance. Surgical treatment consisted of individualized combinations of soft-tissue realignment, quadriceps lengthening, Roux–Goldthwait procedures, and MPFL reconstruction. Statistical analyses evaluated predictors of recurrence and postoperative Lysholm score. **Results:** Thirty-four patients (45 knees; mean age 12 years; 73.5% female) were included. Preoperative Lysholm scores improved from a mean of 73 to 94 postoperatively (*p* < 0.0001). Recurrence occurred in 32.35% of patients and was significantly associated with generalized hyperlaxity (*p* = 0.0041), trochlear dysplasia (*p* = 0.045), and lateral patellar subluxation (*p* = 0.039). Suboptimal postoperative Lysholm scores (<85) were observed in 11.76% of patients, all with recurrence, and were significantly associated with genu valgum (*p* = 0.0011) and patella alta (*p* = 0.036). No significant associations were found for rotational deformities or femoral condyle hypoplasia. **Conclusions:** Habitual patellar dislocation in children is multifactorial, and the likelihood of recurrence increases with cumulative risk factors such as hyperlaxity, trochlear dysplasia, lateral subluxation, patella alta, and genu valgum. Comprehensive preoperative assessment is essential to guide combined, individualized surgical strategies that optimize stability and functional recovery. No single technique is universally curative; rather, tailored multimodal approaches yield the most favorable outcomes.

## 1. Introduction

Habitual patellar dislocation is a distinct form of patellofemoral instability in which the patella consistently displaces laterally during knee flexion and typically reduces on extension. Although less common than acute or recurrent dislocations, habitual dislocation disproportionately affects children and adolescents and may lead to progressive femoropatellar pain, chondral damage, and early degenerative changes if not identified and treated appropriately [1,2].

The condition is multifactorial: major pathoanatomic contributors include trochlear dysplasia, patella alta, and lateralization of the tibial tubercle (commonly quantified as an increased tibial tubercle–trochlear groove distance—TT–TG distance). These structural abnormalities alter the bony containment and the vector of the extensor mechanism, increasing the propensity for lateral translation of the patella. Trochlear dysplasia remains the most frequently identified osseous abnormality in children and adolescents with patellar instability [2,3].

Soft-tissue and dynamic factors are equally important [4]. Generalized ligamentous laxity (evaluated with the Beighton score) and imbalance between medial and lateral stabilizers (for example, vastus medialis obliquus (VMO) insufficiency versus lateral retinacular contracture) significantly raise the risk of habitual and recurrent events, and syndromic connective-tissue conditions (Ehlers–Danlos, Marfan, Down syndrome, etc.) are well-described contributors in the pediatric population [4,5].

Surgical management strategies—ranging from proximal realignment (lateral release, medial plication, quadriceps lengthening) to distal realignment (Roux–Goldthwait, tibial tubercle transfer) and medial patellofemoral (MPFL) reconstruction—must be individualized to the patient’s anatomy [6]. Contemporary evidence indicates that isolated soft-tissue procedures may be insufficient when high-risk osseous features (severe trochlear dysplasia, marked patella alta, or large TT–TG) are present, and that combined procedures are frequently necessary to restore durable stability [7]. Outcomes after MPFL reconstruction and other procedures are generally favorable, but recurrence and persistent instability still occur in a subset of patients [8]. Reported recurrence rates vary across series and systematic reviews, emphasizing the need for careful preoperative risk stratification [8].

Because pediatric patients undergo ongoing skeletal growth and remodeling, standard adult cutoffs (e.g., absolute TT–TG thresholds) may not always apply; therefore, a comprehensive preoperative assessment that integrates clinical examination, growth-stage, and multimodal imaging (radiographs, MRI/CT measurements of TT–TG and trochlear morphology) is necessary to guide the appropriate combination of soft-tissue and bony procedures and to minimize recurrence [1,9].

Against this background, this study aims to identify the demographic, clinical, and imaging factors associated with postoperative recurrence and unfavorable outcomes in pediatric patients with habitual patellar dislocation. A clearer understanding of risk factors may guide personalized surgical strategies and improve long-term functional results and quality of life.

## 2. Materials and Methods

### 2.1. Study Design

This study was designed as a retrospective cohort analysis evaluating demographic, clinical, and imaging risk factors associated with habitual patellar dislocation in the pediatric population. The research protocol was approved by the Institutional Ethics Committee of the “Grigore Alexandrescu” Children’s Emergency Hospital (approval no.37/13 March 2024). Written informed consent was obtained from the parents or legal guardians of all participants. Data were collected from the medical records and imaging archives of patients treated between January 2016 and December 2024 in the Pediatric Orthopedics Department.

### 2.2. Participants

Eligible participants were pediatric patients diagnosed with habitual patellar dislocation, defined as recurrent lateral displacement of the patella during each episode of knee flexion, with spontaneous reduction on extension. Inclusion criteria comprised: positive diagnosis of habitual patellar dislocation, age ≤ 18 years at the time of diagnosis, a minimum clinical and radiological follow-up of 12 months, availability of standard imaging studies (anteroposterior, lateral, and axial radiographs, and MRI or CT when indicated), and signed informed consent.

Exclusion criteria were other forms of patellar instability (post-traumatic, recurrent, permanent, or neuromuscular dislocation), prior knee surgery or fractures involving the tibial plateau or femoral condyles, insufficient imaging documentation, follow-up <12 months, and absence of informed consent.

**Figure 1:**
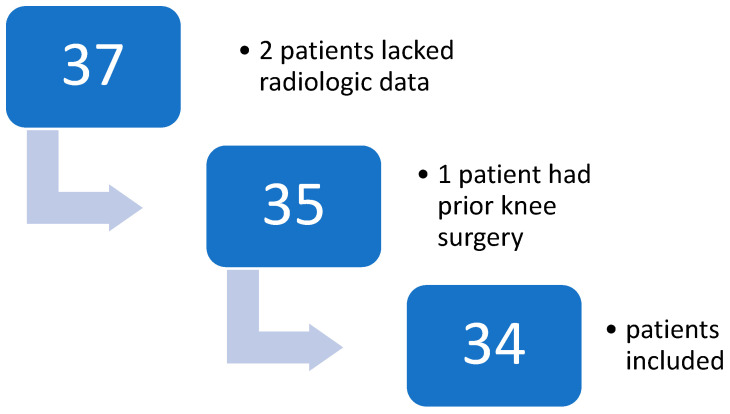
Flowchart of patient selection.

Of the initial 37 patients, 2 had insufficient radiologic data, and 1 had a history of prior knee surgery. Figure 1 illustrates the flowchart of patient selection.

### 2.3. Study Procedure

**Figure 2:**
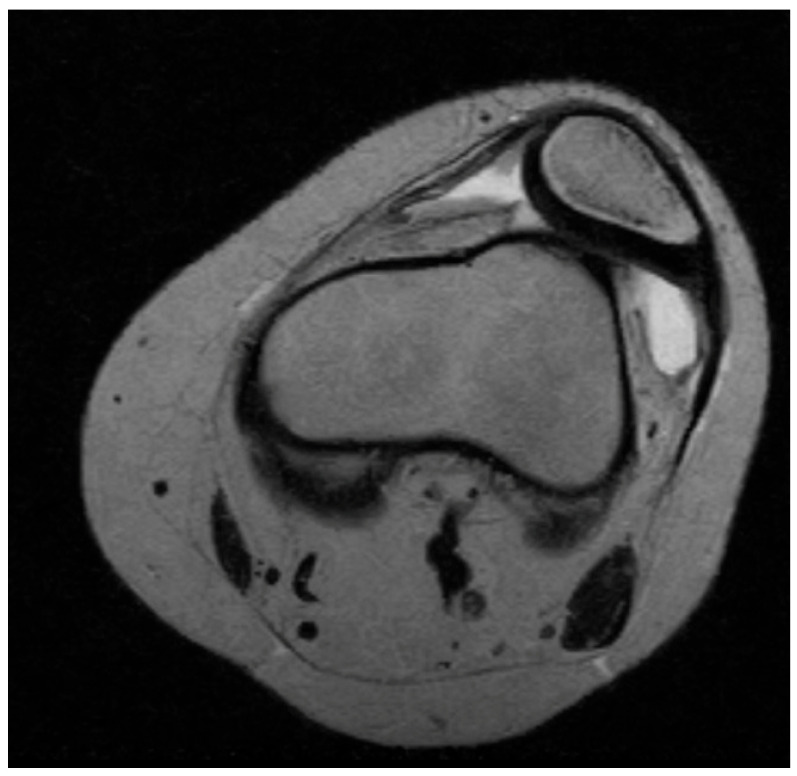
Axial MRI in extension of an 8-year-old girl with habitual patellar dislocation. A patellar subluxation and patellar tilt, as well as trochlear dysplasia, are easily observed.

All patients underwent standardized clinical assessment, including documentation of age, sex, laterality, body mass index (BMI) as demographic data, Beighton hyperlaxity score, and lower limb alignment. Range of motion, quadriceps strength, and presence of apprehension or J-sign were recorded. Radiological parameters were evaluated on standing anteroposterior, lateral, and Merchant or Laurin axial views, as well as MRI scans when available. Figure 2 illustrates an axial MRI view of an 8-year-old girl with habitual patellar dislocation.

**Figure 3:**
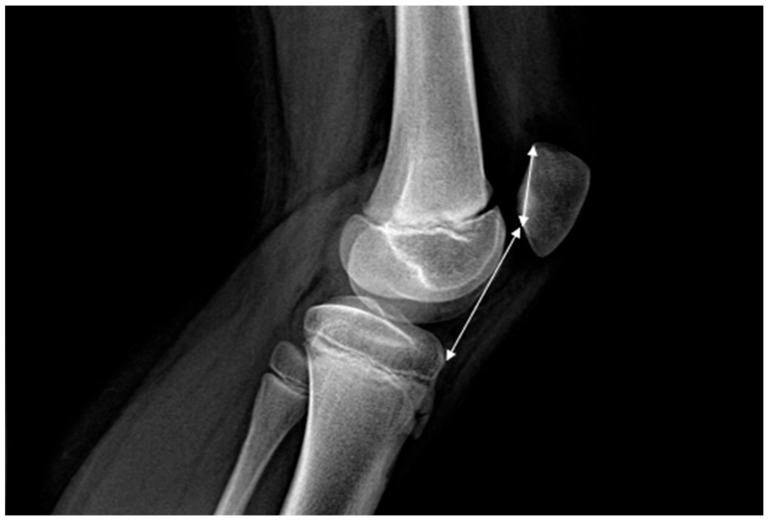
CDI represents the ratio between the length of the patellar articular surface and the distance from the anterior angle of the tibial plateau to the most inferior point of the patellar articular surface, measured on a lateral radiograph with the knee in 30 degrees of flexion.

The following imaging variables were measured: patellar height, assessed using the Caton–Deschamps Index (CDI) with normal values between 0.6 and 1.2, trochlear dysplasia, classified according to Dejour types A–D, patellar dysplasia, according to Wiberg types I–IV, lateral condyle hypoplasia, TT–TG distance which is normal under 15 mm, measured on MRI or CT, sulcus angle normally ranging between 135° and 140°, and lateral patellofemoral angle (Laurin) which normally opens laterally, patellar tilt, knee valgus angle measured on AP ortoleg using hip–knee–ankle angle (HKA), femoral anteversion and tibial torsion, when available [3,10,11,12]. Figure 3 shows the necessary parameters to calculate CDI.

**Figure 4:**
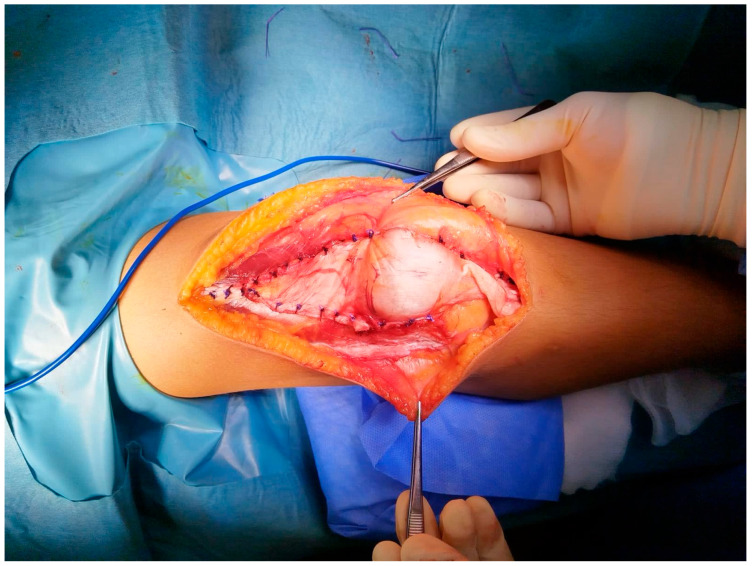
Intraoperative appearance of one of the patients included in the study. A combined four-step technique was employed: (i) release of the lateral retinaculum and plication of the medial retinaculum, (ii) Y-lengthening of the quadriceps tendon, and (iii) patellar tendon transposition via the Roux–Goldwaith technique.

Surgical management was individualized “à la carte” according to patient age, skeletal maturity, and anatomical abnormalities. Bilateral cases were treated in a staged manner, at least 6 months apart. Procedures included soft-tissue realignment (lateral release for patellar tilt, medial plication for medial retinaculum strengthening, or quadriceps lengthening for patella alta), distal realignment for a TT–TG distance > 20 mm (Roux–Goldthwait procedure when physes are open, tibial tubercle transfer when it is fused), and reconstruction of the medial patellofemoral ligament (MPFL) with semitendinosus autograft and interference screws. Figure 4 illustrates an extensive soft-tissue realignment of both the proximal and distal extensor apparatus, incorporating four components: release of the lateral retinaculum and plication of the medial retinaculum, Y-lengthening of the quadriceps tendon, and patellar tendon transposition via the Roux–Goldthwait technique, which consists of a longitudinal split of the patellar tendon, detachment of its lateral half from the tibia, and transposition beneath the medial half, followed by fixation to the tibia with sutures.

All patients experienced a favorable postoperative course without complications. A cast immobilization was maintained for three weeks, followed by a standardized rehabilitation protocol recommended to all patients. Table 1 illustrates the postop protocol.

Functional knee status was evaluated both preoperatively and postoperatively using the Lysholm Knee Scoring Scale, administered as a questionnaire completed by the patients’ caregivers [13]. This scale was selected because it reflects the impact of habitual patellar dislocation—and its surgical correction—on the patient’s quality of life, while also being simple and quick. The Lysholm score comprises eight items (limping, need for crutches, episodes of locking, sensation of instability, pain, swelling, ability to climb stairs, and ability to squat), with a maximum score of 100 points.

### 2.4. Statistical Analysis

Demographic and clinical variables were summarized using descriptive statistics (mean, standard deviation, and range). Each knee was treated as an independent observation, regardless of laterality. Continuous variables (e.g., age, CDI, TT–TG) were analyzed using Student’s *t*-test or Mann–Whitney U test, depending on data distribution. Categorical variables (sex, side, presence of trochlear dysplasia, MPFL rupture) were compared using the Chi-square or Fisher’s exact test. Correlation between anatomical factors and postoperative recurrence was evaluated using Pearson’s or Spearman’s correlation coefficients. Univariable logistic regression was employed to identify independent predictors of postoperative recurrence or suboptimal functional outcomes. A *p*-value < 0.05 was considered statistically significant. Statistical analyses were performed using MedCalc Statistical Software version 20.0 (MedCalc Software Ltd., Ostend, Belgium).

## 3. Results

**Figure 5:**
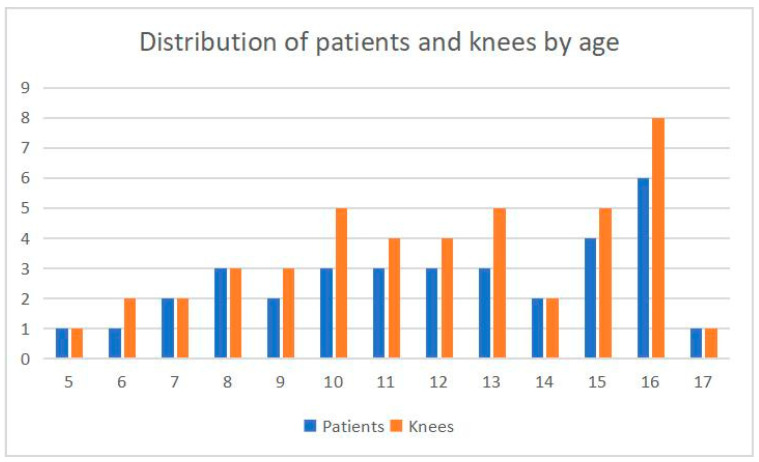
Age distribution of the study cohort. Blue bars represent the number of patients at each year of age, while orange bars represent the number of knees per year of age. The abscissa represents age, and the ordinate represents the number of cases.

The study included 34 pediatric patients (25 girls and 9 boys) aged 5 to 17 years (mean age 12 years) with habitual patellar dislocation, encompassing 45 operated knees as seen in Figure 5.

The cohort was characterized by a predominance of females (73.53%), with no significant differences in age distribution (*p* = 0.1429) or body mass index (BMI; mean, 18.8; *p* > 0.05) between sexes. Bilateral involvement was observed in 50% of patients, with no significant laterality preference (*p* = 0.1673). Generalized ligamentous hyperlaxity (Beighton score) was present in 58.82% of patients, more prevalent in females (*p* = 0.0002).

**Figure 6:**
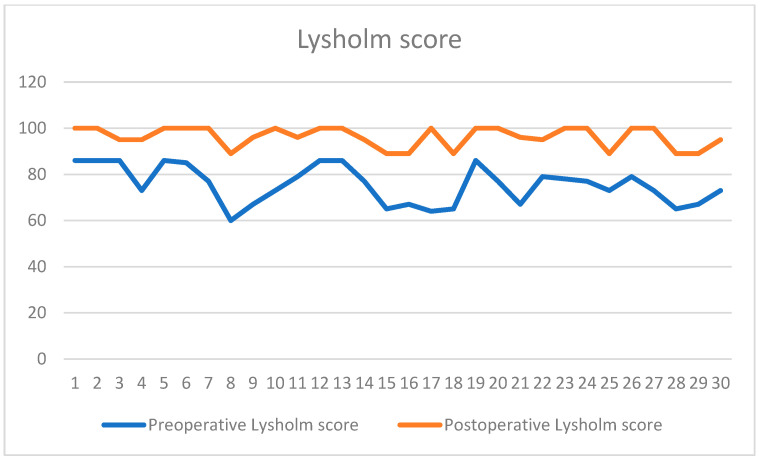
The preoperative and postoperative Lysholm scores are illustrated in the figure above. The preoperative Lysholm score for the study group had a mean value of 73 points, and the postoperative score had a mean value of 94 points.

Preoperative Lysholm scores averaged 73 (range 54–85), improving postoperatively to 94 (range 65–100; *p* < 0.0001 for improvement) as seen in Figure 6.

Postoperative relapse occurred in 32.35% of patients, predominantly in females (*p* < 0.0001), with hyperlaxity as a significant risk factor (81.81% in recurrence group, *p* = 0.0041). As Table 2 illustrates, the Lysholm score was significantly lower in the relapse group.

Suboptimal outcomes (Lysholm score < 85) were seen in 11.76% of patients, all with recurrence (*p* = 0.0024). Surgical interventions combined medial plication and lateral release in all cases, with additional quadriceps lengthening (20%) or Roux–Goldthwait patellar tendon transposition (60%), yielding no immediate complications. Imaging variables were analyzed for associations with recurrence and Lysholm scores, revealing significant links to trochlear dysplasia, lateral subluxation, genu valgum, and patella alta.

Univariable logistic regression analysis was used to identify independent predictors of both the outcomes (recurrence rate and Lysholm score).

### 3.1. Genu Valgum

Genu valgum was identified in 10 of 45 knees (22.22%). In the recurrence group (*n* = 14 knees), it was present in 4 knees (28.57%), compared to 6 of 31 knees (19.35%) without recurrence; this difference was not statistically significant (χ^2^ test, *p* = 0.520, odds ratio [OR] = 1.67). For Lysholm outcomes, genu valgum was observed in 4 of 5 knees (80%) with scores < 85, versus 6 of 40 knees (15%) with scores ≥ 85, showing a significant association (χ^2^ test, *p* = 0.0011, OR = 22.67).

### 3.2. Axial and Rotational Deformities

Femoral anteversion and/or tibial torsion were present in 10 of 45 knees (22.22%). Among knees with recurrence, 3 (21.43%) exhibited these deformities, similar to 7 of 31 knees (22.58%) without recurrence (χ^2^ test, *p* = 0.937, OR = 0.93). In the suboptimal Lysholm group (<85), 1 of 5 knees (20%) had deformities, compared to 9 of 40 knees (22.5%) with scores ≥ 85 (χ^2^ test, *p* = 0.899). No significant associations were found.

### 3.3. Femoral Condyle Hypoplasia

Femoral condyle hypoplasia was noted in 8 of 45 knees (17.78%). It occurred in 3 of 14 knees (21.43%) with recurrence and 5 of 31 knees (16.13%) without (χ^2^ test, *p* = 0.661, OR = 1.42). For Lysholm scores, it was present in 2 of 5 knees (40%) with scores < 85 and 6 of 40 knees (15%) with scores ≥ 85 (χ^2^ test, *p* = 0.178, OR = 3.78, 95% CI [0.54–26.29]). Differences were not statistically significant.

### 3.4. Tibial Tuberosity Lateralization

Tibial tuberosity lateralization measured with the TT–TG distance was evident in 18 of 45 knees (40%). It was more common in the recurrence group (8 of 14 knees, 57.14%) than without recurrence (10 of 31 knees, 32.26%; χ^2^ test, *p* = 0.098, OR = 2.80), though not reaching significance. For Lysholm outcomes, it affected 3 of 5 knees (60%) with scores < 85 and 15 of 40 knees (37.5%) with scores ≥85 (χ^2^ test, *p* = 0.345).

### 3.5. Trochlear Dysplasia

Trochlear dysplasia was the most prevalent factor, present in 29 of 45 knees (64.44%). It was significantly associated with recurrence, occurring in 12 of 14 knees (85.71%) with recurrence versus 17 of 31 knees (54.84%) without (χ^2^ test, *p* = 0.045, OR = 4.94). All 5 knees (100%) with Lysholm scores <85 had trochlear dysplasia, compared to 24 of 40 knees (60%) with scores ≥ 85 (χ^2^ test, *p* = 0.072, OR undefined due to 100% prevalence), approaching but not reaching significance.

### 3.6. Lateral Patellar Subluxation

Lateral patellar subluxation was observed in 25 of 45 knees (55.56%). It was significantly more frequent in the recurrence group (11 of 14 knees, 78.57%) than without (14 of 31 knees, 45.16%; χ^2^ test, *p* = 0.039, OR = 4.45). In the Lysholm analysis, it affected 4 of 5 knees (80%) with scores < 85 and 21 of 40 knees (52.5%) with scores ≥ 85 (χ^2^ test, *p* = 0.244, OR = 3.62).

### 3.7. Patellar Tilt

Patellar tilt was identified in 23 of 45 knees (51.11%). It occurred in 10 of 14 knees (71.43%) with recurrence and 13 of 31 knees (41.94%) without (χ^2^ test, *p* = 0.066, OR = 3.46), approaching significance. For Lysholm scores, it was present in 4 of 5 knees (80%) with <85 and 19 of 40 knees (47.5%) with ≥85 (χ^2^ test, *p* = 0.182, OR = 4.42).

### 3.8. Patellar Height (Patella Alta/Baja)

Patella alta was noted in 25 of 45 knees (55.56%), with patella baja in 1 knee (2.22%). Patella alta was present in 9 of 14 knees (64.29%) with recurrence and 16 of 31 knees (51.61%) without (χ^2^ test, *p* = 0.440, OR = 1.68). It was significantly associated with suboptimal Lysholm scores, occurring in all 5 knees (100%) with <85 versus 20 of 40 knees (50%) with ≥85 (χ^2^ test, *p* = 0.036, OR undefined). The single patella baja case had no recurrence, and the Lysholm score was ≥85.

### 3.9. Caton–Deschamps Index

The CDI was 1.33 (SD 0.28). In the recurrence group, mean CDI was 1.42 (SD 0.31) versus 1.29 (SD 0.25) without recurrence (*t*-test, *p* = 0.109). For Lysholm outcomes, mean CDI was 1.59 (SD 0.32) in the <85 group versus 1.29 (SD 0.24) in the ≥85 group (*t*-test, *p* = 0.052), not statistically significant.

## 4. Discussion

Patellar instability in skeletally immature patients is a multifactorial condition in which clinical and radiological factors interact to determine outcome after surgical treatment. In our cohort of 34 patients (45 operated knees), we observed a pattern of great functional improvement after surgery, but also identified several anatomical and clinical predictors of poor postoperative function or recurrence.

Clinical landmarks

Our data regarding the Lysholm score showed highly significant improvement after surgery and a strong association between recurrence and low postoperative Lysholm (all patients with low postoperative Lysholm had recurrence, and only 23.3% of high-score patients; *p* = 0.0024). These findings reinforce that patient-reported function closely follows mechanical stability: recurrence events reliably produce worse PROMs. Contemporary systematic reviews and cohort studies of MPFL reconstruction and combined approaches in children and adolescents consistently report large, clinically meaningful improvements in Lysholm/Kujala scores after surgery and show that recurrence is the principal driver of worse long-term PROMs [14,15]. Clinically, this supports prioritizing durable stability in the index operation (identifying and addressing the anatomic contributors rather than relying on minimal soft-tissue procedures only) because functional recovery depends on preventing recurrent instability.

In our cohort, generalized hyperlaxity was present in 81.8% of patients who experienced recurrence; this association reached statistical significance (*p* = 0.0041; chi-square). This result matches a growing body of literature that identifies ligamentous laxity as an important risk factor for failure after soft-tissue reconstruction. Studies and reviews note that hypermobility reduces the passive restraint of the capsuloligamentous envelope and may necessitate augmented or combined reconstructions to achieve durable results in children [1,16]. In practice, hyperlax patients should be flagged preoperatively and counseled about higher recurrence risk; surgical planning should consider reinforcing reconstructive choices (e.g., stronger fixation, supplementary procedures) where appropriate.

Radiologic landmarks

Our analysis showed a strong association between genu valgum and low postoperative Lysholm (OR 22.67, *p* = 0.0095 for the Lysholm outcome), while genu valgum was not associated with recurrence per se. This is clinically plausible: a persistent coronal malalignment (valgus axis) increases the lateral vector across the patellofemoral joint, impairs tracking and loading patterns, and can leave patients symptomatic despite medial soft-tissue stabilization. Recent pediatric and biomechanical reports emphasize that coronal malalignment (including genu valgum) is an important modifiable contributor to residual symptoms and failure to return to full function [17,18]. Where significant and symptomatic, corrective osteotomy or guided growth (temporary hemiepiphysiodesis) can be considered in skeletally immature patients as part of an “à la carte” approach [17,18].

Trochlear dysplasia was present in 85.7% of knees with recurrence vs. 54.8% without, producing a significant association in our series (*p* = 0.045; OR ≈ 4.9). This aligns with studies that identify trochlear morphology as one of the strongest predictors of recurrent patellar instability [1,3]. The flat or hypoplastic trochlea provides limited osseous containment in early flexion, increasing reliance on soft-tissue restraints. When dysplasia is high grade, isolated MPFL reconstruction frequently under-addresses the problem, and recurrence rates increase. Severe dysplasia may require trochleoplasty or combined procedures in older adolescents, while in younger children, surgeons often employ combined soft-tissue strategies while deferring bony corrections until skeletal maturity [1]. In our paper, OR (~5) is within the range reported by other authors (commonly 2–5X increased odds), which strengthens the external validity of this finding.

Lateral subluxation occurred in 78.6% of recurrent knees vs. 45.2% of non-recurrent knees in our cohort (*p* = 0.0389; OR ≈ 4.45). Persistent lateral maltracking is described as a mechanical driver of failure after isolated medial soft-tissue repair: it maintains an abnormal lateralizing vector across the patella and overloads the reconstructed medial restraints. Reviews and imaging studies highlight the importance of quantifying patellar translation/subluxation and addressing it (for example, with medialization strategies, or tubercle procedures where skeletally permissible) to reduce recurrence risk [15,19]. In short, our finding supports the current consensus.

All knees that obtained a low postoperative Lysholm score (<85) had patella alta. Patella alta delays trochlear engagement in early flexion, increasing the risk of instability and symptomatic dysfunction. Several pediatric imaging and outcome studies identify patella alta as a contributor to recurrence and worse functional scores, although its independent predictive value sometimes attenuates when adjusted for other factors (trochlear dysplasia, TT–TG) [1,20]. Nevertheless, patellar height remains a clinically useful, measurable parameter and should inform whether additional procedures (such as distalisation of the patellar tendon in skeletally mature patients) or other adjustments are necessary.

Although MPFL reconstruction improves quality of life for patients with patellar instability, as shown in the current study, MPFL reconstruction alone is not sufficient to restore the correct biomechanics of the knee [21].

Across clinical and radiological findings, a clear theme emerges: multiple risk factors frequently coexist, and the presence of more than one (e.g., hyperlaxity + trochlear dysplasia + lateral subluxation or patella alta) increases the likelihood of poor functional outcome or recurrence. Our data show this pattern: isolated soft-tissue reconstruction can work well when anatomical risk is limited, but when significant osseous or alignment issues exist, an augmented or combined strategy (or addressing alignment/growth modulation in children) yields more durable results—an approach echoed by recent “à la carte” treatment algorithms and systematic reviews [15,22].

The study’s retrospective design, modest sample size, and incomplete availability of MRI/CT in some patients (limiting TT–TG measurement) restrict the power for multivariable modeling or the generalizability of specific thresholds. Also, the patient pool is insufficient to correlate each surgical technique or age interval with the outcomes. Future multicentric studies may be useful in contouring guidelines for pediatric patellar instability.

## 5. Conclusions

Habitual patellar dislocation has a multifactorial etiology in which the likelihood of recurrence increases with the accumulation of aggravating factors.

Preoperative assessment of risk factors is essential for selecting combined surgical techniques that ultimately increase the chances of success and reduce recurrence rates.

Habitual patellar dislocation remains a current clinical challenge for which no single definitive procedure exists; instead, a combination of procedures is required to achieve optimal outcomes and facilitate the patient’s reintegration into society.

## Figures and Tables

**Table 1:** Postoperative protocol for the patients included in the study. Postoperative follow-up included clinical and radiological evaluations at 3, 6, and 12 months, and annually thereafter. Outcomes were assessed for recurrence, residual instability, range of motion, and patient-reported satisfaction.

Phase	Time Post-Op	Goals	Activities/Exercises
**Phase I: Protection & Early Motion**	3–6 weeks	Protect, control pain/swelling, maintain patellar mobility, activate quadriceps	-Brace/assistive device as directed
			-Partial weight-bearing
			-Gentle ROM (0–90° flexion)
			-Quadriceps sets, straight-leg raises, ankle pumps
			-Patellar mobilizations
**Phase II: Strengthening & Proprioception**	6–12 weeks	Restore strength, improve neuromuscular control, and normalize gait	-Full weight-bearing as tolerated
			-Closed-chain exercises (mini-squats, leg press)
			-Hip and core strengthening
			-Balance/proprioception training
			-Stationary bike, pool exercises as tolerated
**Phase III: Advanced Strength & Function**	3–6 months	Achieve full function, dynamic control, readiness for sport	-Advanced strengthening (single-leg squats, lunges)
			-Plyometric exercises (if cleared)
			-Functional training, sport-specific drills
			-Objective criteria-based return to running/sport

**Table 2:** Analysis of the relapse-based subgroups indicates that patients with lower preoperative Lysholm scores tend to have correspondingly lower postoperative scores.

	Preoperative Lysholm Score		Postoperative Lysholm Score	
	With Relapse	Without Relapse	With Relapse	Without Relapse
**Mean**	64	78	85	98
**Count**	11	23	11	23
**Standard deviation**	±6.11	±7.48	±9.82	±3.47
***p*-value**	0.000006318		0.000002433	

## Data Availability

The data presented in this study are available on request from the corresponding author.
